# Miscellaney

**Published:** 1896-12

**Authors:** 


### Miscellany.

### ANTITOXIN COLLECTIVE INVESTIGATION (SECOND) AMERICAN PEDIATRIC SOCIETY.

*To the Medical Profession:*

The American Pediatric Society is encouraged to ask the coöperation of physicians in a further collective investigation. Laryngeal diphtheria is believed to furnish a crucial test for antitoxin. The present aim is to ascertain: (1) What percentage of cases of laryngeal diphtheria recover without operation under antitoxin treatment? (2) What percentage of operated cases revover?

The society asks the record of cases of diphtheria involving the larynx, whether operated or not, occurring in private practice in the United States and Canada treated with antitoxin. It is expected that the cases occurring this year will be treated with reliable preparations of the serum, will be treated early and will be given efficient doses. The second report is designed to be a study of cases occurring between the closing of the first report, May 1, 1896, and the closing of the present collective investigation, April 1, 1897.

In order to secure data which shall make the tables complete, circulars containing blanks for ten cases have been printed and are now ready for distribution. It is desired that physicians shall fill out circulars, blanks, as cases occur, not trusting to memory, and shall urge their friends having similar cases to do the same. Circulars can be had by applying to the committee (address below). Several groups of cases in the first investigation arrived too late and were lost to the report. It is desired that circulars as soon as filled (ten cases) be returned to the committee. The collection of cases must close at the end of March, 1897.

For extra circulars (blanks) for returning circulars (filled) and for further information, address the chairman of the committee,

W. P. NORTHRUP, M. D.,
October, 1896. 57 East 79th street, New York, N. Y.

The action of the society upon the (first) report: (1) *Dosage.*—For a child over 2 years old, the dosage of antitoxin should be in all laryngeal cases with stenosis, and in all other severe cases, 1,500 to 2,000 units for the first injection, to be repeated in from eighteen to twenty-four hours if there is no improvement; a third dose after a similar interval, if necessary. For severe cases in children under 2 years, and for mild cases over that age, the initial dose should be 1,000 units, to be repeated as above, if necessary; a second dose is not usually required. The dosage should always be estimated in antitoxin units and not of the amount of serum.

(2) *Quality of antitoxin.*—The most concentrated strength of an absolutely reliable preparation.

(3) *Time of administration.*—Antitoxin should be administered as early as possible on a clinical diagnosis, not waiting for a bacteriological culture. However late the first observation is made, an injection should be given unless the progress of the case is favorable and satisfactory.

---

THE RUSH MONUMENT.—The Rush Monument committee has issued the following circular:

*My Dear Doctor*—The remarkable success of the relatively small body of homeopaths in the United States, in collecting $75,000 for their monument to Hahnemann, to be erected in the City of Washington, ought to make every reputable regular physician in the country keenly alive to the necessity for promptly subscribing to the fund for the long-delayed monument to Benjamin Rush.

The model for the monument to Hahnemann, which has been on exhibition in New York City, has attracted general admiration on account of its great beauty and artistic excellence, which will make it unrivaled as a work of this kind.

The regular medical profession, numbering over 100,000 more than the entire body of homeopaths, has thus far subscribed less than $4,000 towards the projected monument to Dr. Rush, for which the navy department has already generously designated a commanding site in the park fronting the U. S. Naval Museum of Hygiene, where it will be one of the most conspicuous features of the national capitol.

Are the regular physicians of the United States willing that this illustrious signer of the Declaration of Independence and the distinguished medical hero of the Revolution shall be commemorated by an insignificant bust or a mediocre statue, in pitiable contrast with the splendid testimonial at their capitol city to a foreign theorist by a comparatively small body of his misguided followers ? The crowds of visitors to Washington cannot fail to attach a disparaging significance to the spectacle of these two monuments, and we appeal to you to aid the committee in its endeavor to do justice to the memory of this great father of American medicine, pure and undefiled, by sending by return mail to either of the undersigned, as large a contribution as you may be able to make.

ALBERT L. GIHON, M. D.,
*Chairman R. M. C., 8 West 127th Street, New York City.*

GEORGE H. ROHÉ, M. D.,
*Secretary and Treasurer R. M. C., Sykesville, Md.*

W. MURRAY WEIDMAN, M. D.,
*Chairman R. M. Committee for Pennsylvania, Reading, Pa.*

HENRY D. HOLTON, M. D.,
*Treasurer American Public Health Association, Brattleboro, Vt.*

CHARLES McINTIRE, M. D.,
*Secretary-Treasurer American Academy of Medicine, Easton, Pa.*

It is to be hoped that wealthy and philanthropic citizens will contribute to this object with promptitude. We should no longer hesitate to do honor to one of the distinguished fathers of the Republic, who was also a noted physician.

---

CAROID is the name of a new digestive agent that is manufactured by the Charles Roome Parmele Company, of New York. Prof. R. H. Chittenden, physiologic chemist at the Sheffield scientific school of Yale University, has published the results of his study of the digestive power of caroid as contrasted with papoid and commercial papine. He gives a detailed report of each experiment relating to proteolytic action, six in all, and in relation to their starch digesting power. These are in every instance favorable to caroid, which, Prof. Chittenden says, shows throughout very much greater amyolytic power than the other preparations; also that a like superiority over proteid matter is exhibited. Further information regarding this new agent can be obtained of the manufacturers, 36 Platt street, New York.

---

THE effort being made by Mr. Geo. E. Livingstone, manager of the Press Publishing Co., in creating interest among local physicians on the delinquent debtor question is likely to culminate in the production of a very valuable book of local reference costing a mere trifle. It is about time that the physicians united in supporting something of this kind, and we endorse this scheme as being the simplest and best yet proposed and one almost absolutely void of any objectionable features.

---

PEROXIDE OF HYDROGEN, Hydrozone and Eye Balsam is the title of Prof. Marchand's book on the therapeutical uses of those preparations, the eleventh edition of which has lately appeared. It contains much useful information relating to these important chemical products, is a book of 216 pages and will be sent free to any physician who mentions this journal in connection with his request. Address Charles Marchand, 28 Prince street, New York.

---

EVERY subscriber in arrears received a statement last month, and we are happy to announce that a large number were not satisfied with paying the amount due, but enclosed sufficient to credit their accounts two or three years in advance. A five dollar bill in payment of a debt of but one or two dollars is a handsome way of expressing appreciation of any enterprise in times like these. You have our thanks.— *Ohio Medical Journal*, November, 1896.

---

THE Arlington Chemical Co., on its advertising page in this issue, announces the winners in its $600 prize contest of last year. The personnel of the committee of award, with Dr. Frank P. Foster at its head, was an absolute guarantee of impartiality in the distribution of the prize money. The liberal spirit that has characterised this company is commendable.

